# Supplementary material for: Double-Layer MWCNTs@HPPS Photothermal Paper for Water Purification with Strong Acid-Alkali Corrosion Resistance
Source: Membranes (Basel). 2022 Nov 29;12(12):1208. doi: 10.3390/membranes12121208 (PMC9782072; doi:10.3390/membranes12121208)
Supplement: Supplementary file 1 [file membranes-12-01208-s001.zip › membranes-1908458-supplementary.pdf]

Supporting Information

Double-layer MWCNTs@HPPS photothermal paper for water purification with strong acid-alkali corrosion resistance

Yuan Gao [1,*], Guoqing Jin [1], Shuaishuai Wang [2], Lihua Lyu [1], Xinghai Zhou [1] and Chunyan Wei [1,*]

1 School of textile and materials engineering, Dalian Polytechnic University, Dalian 116034, China.
2 Shandong Chambroad Holding Group Co., Ltd., Binzhou 256500, China.
* Correspondence: gaoyuan@dlpu.edu.cn; Tel.: +411-8631-8763 (Y. G.)

Experiment Sections

Preparation of PPS solid solution powders

PPS solid solution powder was prepared by thermally induced phase separation (TIPS) [1, 2], and the detailed step was performed as follows: PPS, BP and DBP with a weight ratio of 19 %: 65 %: 16 % were mixed together in a three-neck flask at certain temperature. When homogeneous solution was formed and no bubbles existed, the solution was quickly poured into liquid nitrogen for solidification, and then the obtained solid was smashed in a high-speed pulverizer at 24000 r/min for 1min to obtain the PPS solid solution powder.

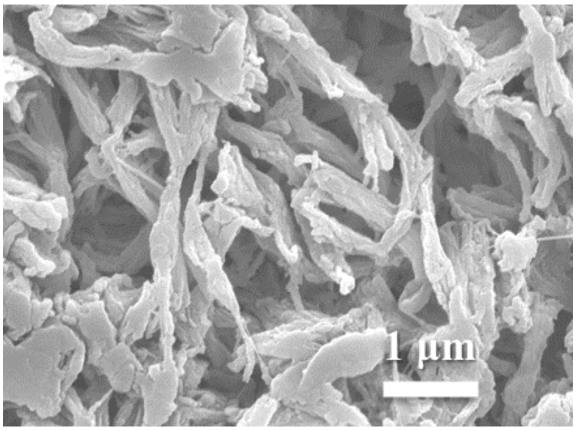

Figure S1. SEM imagine of PPS porous resin structure of 18 % PPS solid solution powder.

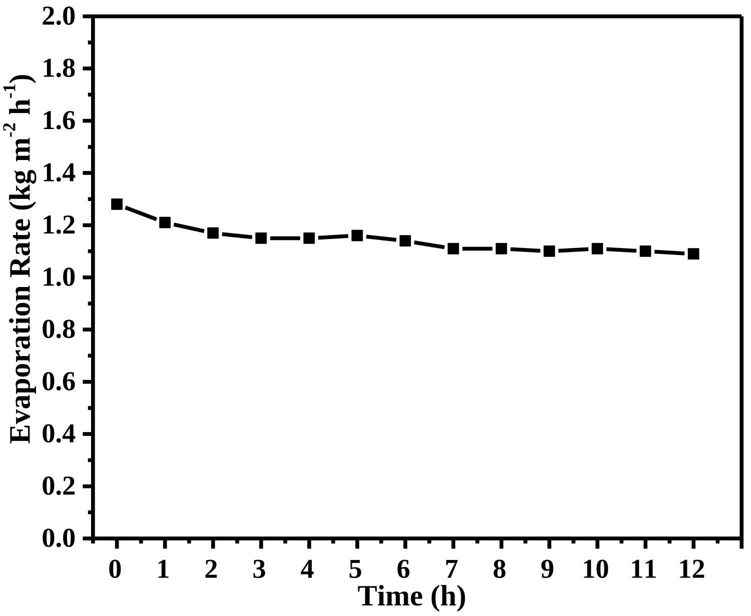

Figure S2. Long-term stability of prepared MWCNTs@HPPS paper with 0.2 g/100 mL EC solution under 1.0 sun irradiation for 12 hours.

Reference

1. X. Wang, Z. Li, M. Zhang, T. Fan, B. Cheng, Preparation of a polyphenylene sulfide membrane from a ternary polymer/solvent/non-solvent system by thermally induced phase separation, Rsc Advances, 7 (2017) 10503-10516.

2. Y. Gao, Z. Li, B. Cheng, K. Su, Superhydrophilic poly(p-phenylene sulfide) membrane preparation with acid/alkali solution resistance and its usage in oil/water separation, Separation & Purification Technology, (2017) 262-270.
